# Effect of fluralaner on the biology, survival, and reproductive fitness of the neotropical malaria vector *Anopheles aquasalis*

**DOI:** 10.1186/s12936-023-04767-0

**Published:** 2023-11-07

**Authors:** João Arthur Alcântara, Francys Sayara Andrade de Araújo, Andréia da Costa Paz, Rodrigo Maciel Alencar, Berta Yoná de Albuquerque Caldas, Raquel Soares Maia Godoy, Marcus Vinicius Guimarães Lacerda, Gisely Cardoso de Melo, Wuelton Marcelo Monteiro, Vanderson de Souza Sampaio, Nágila Francinete Costa Secundino, Ana Paula Marques Duarte, Rosa Amélia Gonçalves Santana, Paulo Filemon Paolucci Pimenta

**Affiliations:** 1 Programa de Pós-graduação em Medicina Tropical–Universidade do Estado do Amazonas/Fundação de Medicina Tropical Dr. Heitor Vieira Dourado (PPGM–UEA/FMT-HVD), Manaus, Brazil; 2 Departamento de Ensino e Pesquisa–Fundação de Medicina Tropical Dr. Heitor Vieira Dourado (DENPE/FMT-HVD), Manaus, Brazil; 3 Programa de Pós-Graduação Em Ciências da Saúde, FIOCRUZ–Belo Horizonte, Belo Horizonte, Minas Gerais Brazil; 4 Instituto René Rachou–Fundação Oswaldo Cruz–(IRR–Fiocruz Minas), Belo Horizonte, Brazil; 5 Instituto Leônidas e Maria Deane – Fundação Oswaldo Cruz – (ILMD – Fiocruz Amazônia), Manaus, Brazil; 6 University of Texas Medical Branch (UTMB), Galveston, USA

**Keywords:** Malaria, Neotropical vector, *Anopheles aquasalis*, Fluralaner

## Abstract

**Background:**

Reducing mosquito abundance or interfering with its ability to support the parasite cycle can help to interrupt malaria in areas of significant risk of malaria transmission. Fluralaner is a safe and effective drug for veterinary use indicated for the treatment against fleas and ticks which acts as an antagonist of chloride ion channels mediated by γ-aminobutyric acid (GABA), preventing the entry of these ions into the postsynaptic neuron, leading to hyperexcitability of the postsynaptic neuron of the central nervous system of arthropods. Fluralaner demonstrated insecticidal activity against different insect species.

**Methods:**

The study aimed to evaluate the effects of fluralaner on the biology, survival, and reproductive fitness of *Anopheles aquasalis*. The following lethal concentrations (LC) were determined for *An. aquasalis*: LC_5_ = 0.511 µM; LC_25_ = 1.625 µM; LC_50_ = 3.237 µM.

**Results:**

A significant decrease (P < 0.001) was evident in the number of eggs, larvae, and pupae in the group exposed to a sublethal dose of fluralaner when compared to a control group (without the drug). Using blood from dogs after administration of fluralaner, it was observed that the drug causes 100% mortality in *An. aquasalis* in less than 24 h after feeding; this effect remains even after 90 days in all samples.

**Discussion:**

Fluralaner showed the same result for up to 60 days, and after that, there was a slight reduction in its effect, evidenced by a decrease in the percentage of dead females; however, still significant when compared to the control group.

**Conclusion:**

Fluralaner affects the biology and reduction of survival in *An. aquasalis* in a lasting and prolonged period, and its fecundity with lower dosages, is a strong candidate for controlling disease vectors.

**Supplementary Information:**

The online version contains supplementary material available at 10.1186/s12936-023-04767-0.

## Background

Malaria is an acute febrile infectious disease caused by the protozoa infection of the genus *Plasmodium*, whose transmission in humans occurs by bites of *Anopheles* vectors. In 2021, more than 247 million estimated cases, representing an increase of 2 million cases compared with 2020, and 619,000 deaths estimated, a slight decline compared with 2020, were recorded worldwide [1]. The disease is considered a major global health problem, mainly affecting populations from subtropical and tropical areas of Africa, Asia, and the Americas, including Brazil, which is almost exclusively restricted to the Amazon region. The Amazon is a vast biome that spans eight developing countries (Brazil, Bolivia, Ecuador, Colombia, Venezuela, Guyana, and Suriname) with a high risk of malaria transmissions due to environmental factors favouring the development of anophelines: high temperatures, relative humidity above 80%, frequent rainfall, and dense vegetation around the breeding sites and dwellings [2, 3]. In addition, physical topographic changes, such as new development projects, dams and road construction, mining, reforestation, deforestation, and commercial plantations, contribute to the increase in malaria cases. The Amazon region has 99.8% of annual malaria cases of Brazil, with the leading parasite, *Plasmodium vivax* [4–7].

The time required for the *Plasmodium* life cycle to be completed in *Anopheles* vectors to reach the salivary gland and be transmitted to a new vertebrate host ranges from 10 to 21 days, depending on the vector-parasite pairs and environmental conditions [8, 9]. The knowledge of this parasite developmental period in the mosquito is primordial for vector control, such as reducing the mosquito abundance and interfering with vector competence to diminish or interrupt malaria transmission.

Insecticides represent an essential and affordable method for reducing malaria transmission rates. Between 2000 and 2015, the reduction in the number of malaria cases and deaths by 50% was closely attributed to insecticide-based vector control, such as insecticide-treated nets (ITNs) and indoor residual spraying (IRS) [10, 11]. These processes are documented as an effective way to control malaria and have been adopted globally by the World Health Organization (WHO) [12]. However, insecticides have proven ineffective in reducing mosquito vectors and malaria transmission. Studies indicated resistance profiles in several anopheline species in different malaria-endemic areas [13, 14]. Between 2010 and 2020, according to the WHO, of the 88 malaria-endemic countries, 78 (88.6%) reported resistance to at least one class of insecticide: 29 (33%) detected resistance to pyrethroids, organochlorines, carbamates, and organophosphates in different sites and 19 (21.6%) confirmed resistance to all categories in at least one location and at least one vector [1].

The use of drugs with endectocide action, such as ivermectin, has gained strength in the context of malaria reduction. A number of studies have been carried out to determine the effectiveness of this drug in the intervention of vectors at different stages of their development [15, 16]. After oral administration in humans, ivermectin can kill mosquitoes and malaria parasites [17–21]. However, ivermectin has a reduced half-life and bioavailability [22], which may make it necessary to use the drug at short intervals in mass use.

Fluralaner is a molecule of the isoxazoline class, which acts as an antagonist of chloride ion channels mediated by the neurotransmitter γ-aminobutyric acid (GABA), preventing the entry of these ions into the postsynaptic neuron, leading to hyperexcitability of the postsynaptic neuron of the central nervous system of arthropods [23]. Fluralaner is a chewable tablet, is therefore easy to administer, and has high efficacy with activity exceeding 12 weeks after oral administration [24, 25]. The drug has higher selectivity for arthropod neurons, being high selectivity against fleas and ticks, and it has been proven to be a good safety profile in vertebrates.

The fluralaner insecticidal activity has been demonstrated against several disease vectors [23–25]. The insecticidal action was evaluated against *Phlebotomus perniciosus*, responsible for transmitting *Leishmania infantum.* A 100% efficacy was evident after 30 days, and this efficacy remained high after 84 days, with more than 50% mortality after 48 h of the feeding experiment [26].

Fluralaner also has insecticidal activity effective against several other species, such as *Culex pipiens*, *Rhipicephalus microplus*, *Ctenocephalides felis*, *Drosophila melanogaster*, *Rhipicephalus sanguineus*, *Lucilia cuprina,* and *Ornithodoros moubata*. In some of these, the effect was also seen in larval stages [27, 28].

Recently, the insecticidal activity of fluralaner has been evaluated against some New World vectors, such as *Lutzomyia longipalpis*, a vector of *Leishmania infantum*. The drug’s effects can last for 5 months with 100% effectiveness, declining to 72.5% in 6 months [30]. In *Triatoma brasiliensis*, the vector of *Trypanosoma cruzi,* the drug had an efficacy of 100% until the 7th month after treatment, remaining at more than 50% after 9 months [30].

Studies have shown the efficacy of fluralaner against *Anopheles stephensi* and *Anopheles gambiae*, *Aedes aegypti,* and *Phlebotomus argentipes* [27]. The effect against a Neotropical vector has recently been described, where it was possible to see the action on fluralaner on the mortality of *Anopheles albimanus* [31]*.* Still, the effect of fluralaner against anopheline vectors in malaria-endemic Brazilian regions has yet to be explored.

Given the permanent risk and problems caused by malaria in Brazil, especially in the Amazon region, associated with the fact that strategies to reduce the vector population through the development of drugs with easy-to-use and long-lasting insecticidal action are gaining incentive, this study aims to evaluate the effects of fluralaner on mortality, survival and reproductive capacity of *Anopheles aquasalis*, the primary coastal malaria vector in the Americas.

## Methods

### Inclusion and collection of samples

Initially, 16 healthy individuals over 18 years of age and of both sexes, who had not used any drug with a proven effect on the mortality of *Anopheles*, were recruited. From each participant, 9 mL of blood was collected, and the blood samples were used in experiments that aimed to define the lethal concentration of the drug and evaluate the effect on the reproductive capacity of *An. aquasalis*, as previously described [32].

Healthy dogs (n = 4) American Pit Bull Terriers (2 males and 2 females) recognized breed by veterinaries, aged 2 years, with an average weight of approximately 34 kg (32.8–35.2) with a veterinary prescription for using fluralaner were included in this study. The following conditions excluded the dogs: (1) pregnant or lactating female; (2) age less than 8 weeks old and with body weight less than 2 kg; (3) That had pancreatic, hepatic, and cardiovascular dysfunctions and (4) That have used any insecticide-acting drug at 1 year before [33]. During this study, the general health status of the dogs was monitored.

The owners of the dogs were oriented about this study. They agreed with the collection of 9 mL of venous blood from the jugular vein or cephalic vein by a veterinarian over different time intervals after the ingestion of the drug: (i) before drug ingestion (BDI-control group), (ii) 24 h post-ingestion (HPI 24), (iii) 7d post-ingestion (DPI 7), (iv) 14d post-ingestion (DPI 14), (v) 21d post-ingestion (DPI 21), (vi) 28d post-ingestion (DPI 28), (vii) 60d post-ingestion (DPI 60) and (viii) 90d post-ingestion (DPI 90).

### Drug acquisition

Fluralaner powder (pure compound) was obtained from Byorbyt (https://www.biorbyt.com/—Cambridge, UK, England) and used to define the lethal concentrations and the effect on reproductive capacity. For the experiment to evaluate the impact on survival after treatment, we used chewable tablets of fluralaner (Bravecto^©^) purchased in stores or pharmacies specializing in selling veterinary products. The dose used was adjusted according to the animal's weight and label recommendation (25–56 mg/kg body weight) (MSD Saúde Animal, SP, Brazil).

### Mosquito colony

*Anopheles aquasalis* mosquitoes were obtained from the Entomology Department of Fundação de Medicina Tropical Dr. Heitor Vieira Dourado (FMT-HVD). The mosquitoes were maintained at 26–27 °C, 70–80% relative humidity, and 12/12 light/dark photoperiod. Larvae were fed commercial fish food (Tetramin Gold^©^), and adults received 10% sucrose solution ad libitum. Females 3–5 days after emergence were used in the experiments.

### ***CL***_***50***_*** estimates***

Initially, the fluralaner powder was dissolved in dimethyl sulfoxide (DMSO) at 10 mg/mL concentration and diluted 1 to 10 in phosphate-buffered saline (PBS). This solution was divided into smaller aliquots containing 100 µL each. A used aliquot was thawed for each experiment, and 900 µL of Iscove’s modified Dulbecco’s medium (IMDM) was added. From there, serial elution (1:100) were performed to obtain different concentrations, as described previously [34–36]. The lethal concentration (LC) assessment was performed by adding the different concentrations from 0.025 to 2 ng/mL) (Additional file 1: Figure S1). These concentrations were added to 1 mL of human blood maintained at 36 °C and offered through membrane feeding assay (MFA) to *An. aquasalis* females for around 30 min. Each experiment had 5 groups of females (150 females each) fed with blood containing different types of concentrations (group FLU), accompanied by control without the drug (group CON) and another with DMSO (group DMSO) of a value equal to the highest concentration of the drug used in that same experiment. Fully engorged females were transferred to rearing cages and kept in an insectary under the same conditions described previously. After 24 h, the number of dead females was recorded to evaluate the mortality rates for each drug concentration.

### Effects on reproduction capacity

Around 150 females were fed with a sub-lethal concentration (LC_5_) of the drug determined according to the previous phase of this study (Additional file 2: Figure S2). After blood feeding, 30 engorged females were transferred to water containers with filter paper for oviposition and kept in an insectary under the same conditions used in the mosquito colony for three days. Afterward, the eggs were transferred to basins to complete their developmental stages. The number of eggs, larvae, and pupae was recorded using the average counting results of three independent researchers. Besides the drug at a low concentration (group FLU), each experiment had a control containing blood without the drug (group CON) and another with blood containing DMSO (group DMSO) at the same concentration used in the FLU group.

### Effects on survival after treatment with fluralaner

Immediately after the first blood collection, fluralaner was administered to the dog, and its total ingestion was confirmed. The exact collection logistics were used on the other days. More details about blood samples and the preparation for membrane blood-feeding assay (washing and removing plasma) are described in the Additional file 3: Text Information.

The collected blood was divided into two parts: one was offered to the females during the first experiment (control), the other was centrifuged, and plasma was stored at -80º C. The remaining red blood cell pellet was reconstituted with the plasma obtained in the first collection without the drug (Drug-free group) [34].

Each experiment had three groups with approximately 150 females, one of which was reserved for drug-free samples. These samples were offered through membrane blood-feeding assay at 36 ºC for 30 min. The fully engorged females were transferred to cages and kept in an insectary, and after 24 h, the number of dead females was recorded.

### Data analysis

To assess the effects of the drug on mosquito survival, Mantel-Cox log-rank test was used. A non-linear mixed model with probit analysis was applied to estimate the in vitro values of LC_5_, LC_25_, and LC_50_. Experiments of lethal concentration with mortality higher than 10% in control were discarded, and mortality lower than this value was corrected by the Abbot formula [37].

Differences between the control and LC_5_ regarding the effects of Fluralaner on fecundity, egg hatching rate, and the number of pupae were estimated by a two-sample Wilcoxon rank sum test (Mann–Whitney) since the sample was not assumed to be normally distributed by the Shapiro–Wilk test.

Efficacy in the dog experiments was assessed by comparing the number of dead mosquito females in the groups with the drug over time versus without the drug (BDI). Differences between groups on different days were determined using Fisher’s test [29, 38] with a two-tailed normality test between groups considering significant differences of P ≤ 0,05.

R studio software (v. 2022.07.0) was used for all analyses. R: A language and environment for statistical computing. R Foundation for Statistical Computing, Vienna, Austria. URL: https://www.R-project.org/) and graphs in GraphPad Prism 8 (v.8.0.2) (GraphPad Software, Inc., San Diego, California. URL: https://www.graphpad.com/company).

## Results

### ***LC***_***50***_*** estimates***

Through 12 experimental replicates, 3121 females were exposed to various concentrations of Fluralaner ranging from 0.025 to 2 ng/mL (Additional file 4: Table S1).

The lethal concentrations were estimated according to the values expressed in Table 1. For *An. aquasalis*, the lethal concentrations (LC) of 5%, 25%, and 50% were, respectively: LC_5_ = 0.511 nM [95%CI 0.276–0.720]; LC_25_ = 1.625 nM [95%CI 1.298–2.016]; LC_50_ = 3.237 nM [95%CI 2.536–4.780]; without significant differences between the groups and control (without the drug: n = 662; and with DMSO: n = 570) (Additional file 5: Figure S3).

**Table 1:** Lethal Concentrations (LC) of Fluralaner for *An. aquasalis*

LC (%)	ng/mL [CI 95%]	nM [CI 95%]
5	0.2839 [0.1539–0.400]	0.5110 [0.2770–0.7200]
10	0.4536 [0.2976–0.5865]	0.8165 [0.5357–1.0557]
15	0.6062 [0.4407–0.7552]	1.0912 [0.7933–1.3594]
20	0.7542 [0.5825–0.9288]	1.3576 [1.0485–1.6719]
25	0.9033 [0.7214–1.1203]	1.6259 [1.2985–2.0166]
30	1.0575 [0.8569–1.3393]	1.9034 [1.5423–2.4107]
35	1.2202 [0.9903–1.5933]	2.1963 [1.7825–2.8680]
40	1.3950 [1.1245–1.8902]	2.5111 [2.0240–3.4024]
45	1.5862 [1.2628–2.2398]	2.8551 [2.2730–4.0316]
50	1.7989 [1.4090–2.6558]	3.2380 [2.5362–4.7805]
99	32.0866 [13.4426–169.2399]	57.7559 [24.1966–304.6319]

*LC* Lethal concentration

### Effects on reproductive fitness

**Fig. 1:**
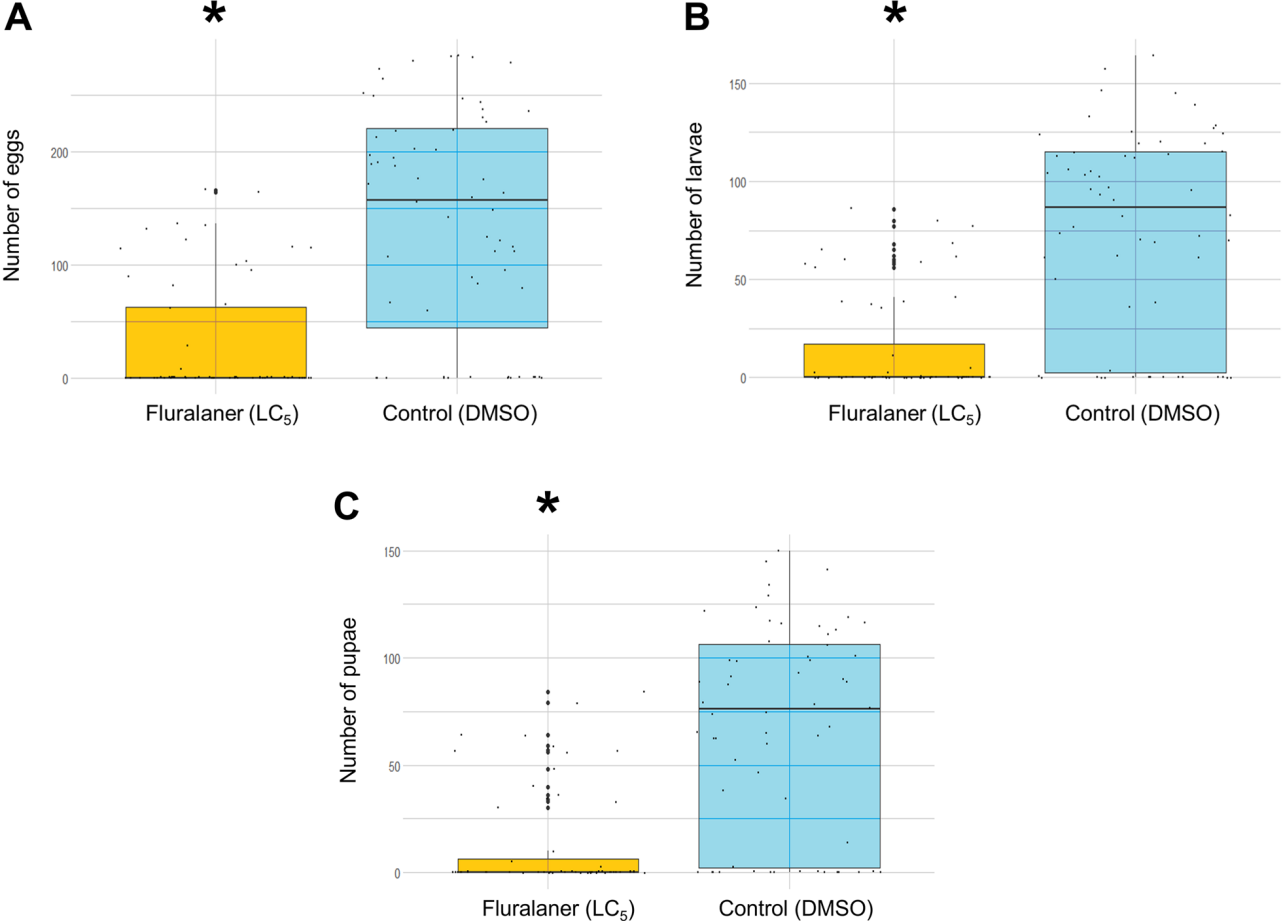
Effects of Fluralaner on the reproductive fitness of *Anopheles aquasalis.* **A** Effect on egg numbers; **B** Effect on larvae numbers; **C** Effect on pupae numbers

*Anopheles aquasalis* females exposed to a low concentration of fluralaner (LC_5_ = 0.511 nM [95% CI 0.276–0.720]) showed alteration in their ability to reproduce. A total of 180 females were fed and placed into forced lay (Additional file 6: Table S2). There were no significant differences between the CON (n = 60) and DMSO (n = 60) groups, so the DMSO group was used in comparison with the drug (FLU, n = 60). After normality and Mann–Whitney tests of the samples, a significant (P < 0.001) decrease in the number of eggs was evident in the FLU group (Median = 0 [IQR = 0–62. 75]) (Fig. 1A), in the number of larvae (Median = 0 [IQR = 0–17] (Fig. 1B), and in the number of pupae (Median = 0 [IQR = 0–6.25] (Fig. 1C) when compared to the DMSO group (eggs—median = 157 [IQR = 44.25–220.75] / larvae—median = 86.5 [IQR = 2.25–115] / pupae—median = 76 [IQR = 2.25–106.5]).

### ***Effects on survival after treatment with fluralaner (Bravecto ***^***®***^***)***

**Fig. 2:**
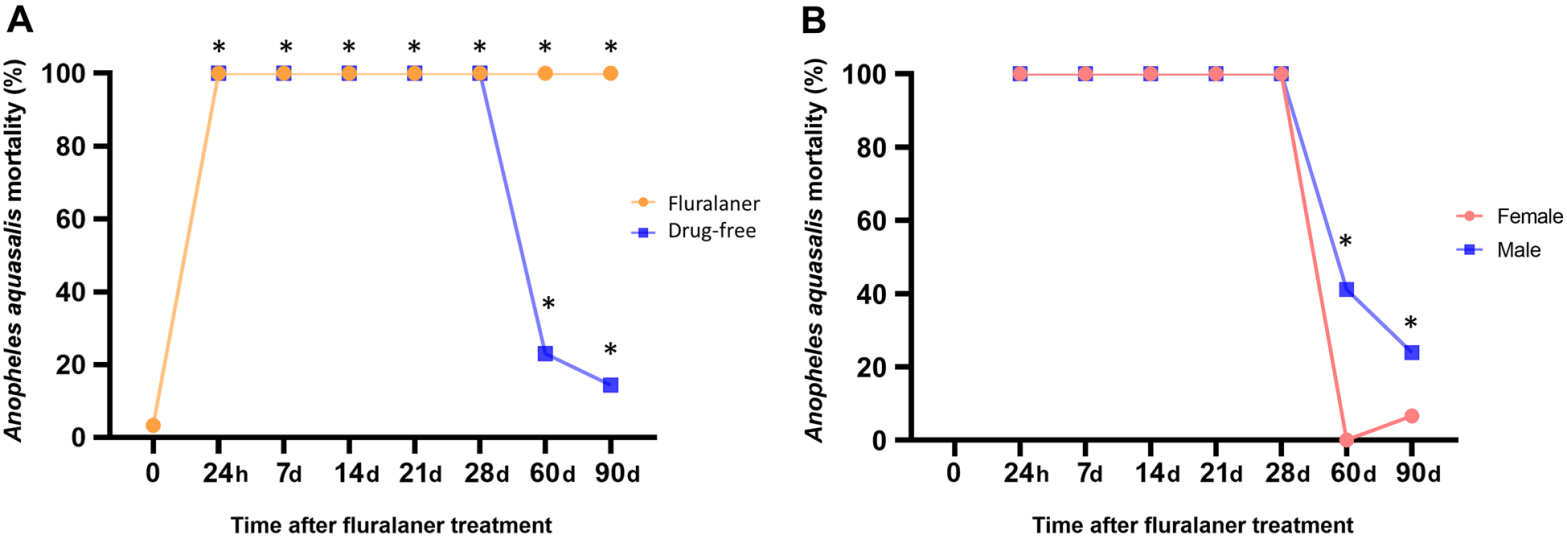
Mortality of *Anopheles aquasalis* (%) over 90 days. **A** After feeding with blood from dogs treated with Fluralaner and after feeding with reconstituted blood (Drug-free). **B** Comparison of mortality after feeding reconstituted blood (Drug-free) of male and female dogs. Data represent two-tailed testing, and Fisher's exact test was used. Asterisk (*) indicates a significant difference between groups at P < 0.05

*Anopheles aquasalis* females fed with blood from the dogs in BDI showed a 100% mortality in all collections maintained throughout 90 days, with all deaths occurring within 24 h after membrane feeding assay (MFA) (Fig. 2A). Interestingly, the drug-free group also showed significant mortality over time (100%), decreasing 60 days (23.5%) and 90 days (14.34%) after MFA. Still, when compared to the mortality of blood-fed females from the control group (BDI) (3.28%), there was a significant difference (P < 0.05) in this group (Additional file 7: Table S3).

There was no difference in mortality between *An. aquasalis* females after MFA of blood from male and female dogs treated with fluralaner. Interestingly, in the fluralaner-free group, after 60 days, a significant difference (P < 0.05) was observed when comparing the sexes of the dogs (Fig. 2B). After the dogs ingested the drug, no adverse manifestations were presented throughout the study period.

## Discussion

Malaria control through the reduction of mosquito vectors has been an effective strategy over the years, especially in endemic areas around the world, such as the Amazon region, where environmental factors favor the maintenance and proliferation of the disease. Vector control and disease prevention using oral insecticides have been presented as innovative strategies. These systemic insecticides are drugs applied directly to vertebrate blood to kill mosquitoes that take a blood meal. Today, most researchers are focusing on Ivermectin, a drug extensively used for river blindness that has recently been proposed for malaria control [32, 34, 35]. However, there is a concern that a possible over-dependence on a single drug will repeat past experiences like the rapid spread of pyrethroid resistance in malaria mosquito vectors. Diversifying the arsenal of systemic insecticides used for mass drug administration would improve this strategy’s sustainability.

Here it was investigated fluralaner, used to treat and prevent fleas and tick infestation in dogs and cats, for malaria vector control through experimental work developed with dogs and observing the ingested drug efficacy on the *An. aquasalis*, an important Neotropical vector. Fluralaner meets several significant criteria, such as a broad safety profile, proven activity against several vectors, and prolonged effect, which can be administered in a single dose, facilitating mass administration and reducing logistical costs. Fluralaner can be a candidate for blocking vector-borne diseases in humans when used as a veterinary parasiticide, including in malaria-endemic regions.

Fluralaner was shown to have significant lethal activity on *An. aquasalis* with an LC_50_ of 3,23 nM (lethal concentration for 50% mortality) 24 h after ingesting dog blood containing the drug. This value is much lower when compared to LC_50_ of other vector species, such as *An. stephensi* (56. nM), *An. gambiae* (33.2 nM), *Ae. aegypti* (34.3 nM), *Cx pipiens* (92.5 nM), *Ph. argentipes* (575.4 nM) and *L. longipalpis* (1,183 nM) [27].

Each species intrinsic characteristics may explain the difference between these distinct LCs. The modulator targets GABA, ionotropic receptors, in which the localization sites of the isoxazoline molecules are located and have different locations in particular insects, including mosquitoes [39–41]. The mechanisms that allow understanding of the interference in the vectors’ physiological activities due to the drug’s action on the nervous system are still being explored [42].

This study is the first one to determine the effect of fluralaner in a Neotropical Brazilian malaria vector considering the LC. It used a different methodology from other studies to recognize the drug effect more accurately [19, 32]. Fluralaner was used in pure powder form, which may have been a determinant to identify the lower value of LC sufficient for effectively indicating the substantial blockage of GABA and L-glutamate gated chloride channels causing the mortality of the *An*. *aquasalis*. The low values determined here are more than suitable to cause a lethal effect on this malaria vector even though it is known that much higher maximum concentrations of the drug are presented in the bloodstreams of dogs [24], cats [43], and cattle [31].

Moreover, the values found here are also well below the safety profile of a possible safe human dosage, where 260 mg Fluralaner is indicated, exceeding the CL_99_ not only shown here for *An. aquasalis* (57.7559 nM), but also for several other insect species [27]. It is worth emphasizing the low selectivity of the drug for GABA receptors in mammals compared to insects [23], which may encourage future research on the insecticidal action of fluralaner.

Many insecticides act on the developmental stages of the mosquito vector life, interfering with its ability to reproduce [31, 32], locomotion [35], vectorial capacity, and other factors that regulate the transmission of diseases [44, 45]. Ivermectin in sub-lethal dosages decreased the reproductive ability of *An. aquasalis* [32]. This study using a similar methodology revealed that fluralaner has a similar effect on this mosquito. The concentration of 0.511 nM (equivalent to LC_5_) significantly decreased the number of all developmental stages of the *An. aquasalis* (eggs, larvae, and pupae) and reduced the number of adult mosquitoes.

Considering the pharmacokinetic data on the effect of fluralaner, the dosage, even if non-lethal, would be working after a long time of direct action of the drug [24, 43]. The drug has a long-lasting effect on disease vector mortality and can interfere with other aspects of vector competence, such as the mosquito reproductive capacity shown here. Also, it is understood that even if the drug is in low concentrations, it can still have a significant effect to the point of impacting malaria transmission [46, 47].

The administration of insecticidal drugs to cattle to reduce malaria has been used and indicated for disease control in field regions [48]. Recently, fluralaner has been explored for this purpose [31]. The authors showed that while fluralaner significantly affected mortality in *An. albimanus* and *An. stephensi*, the drug did not affect reproductive capacity at sub-lethal doses. They pointed out that besides the fact that there are still no specific formulations of fluralaner for other animal species (such as cattle here), there needs to be more information regarding the pharmacokinetics of the drug in other species, which may interfere with the results. Meyers et al*.* [49] highlighted several differences between species within the genus *Anopheles* on physiological aspects of fluralaner*.* Differences were found in the midgut absorption, in the detoxification processes of the mosquitoes, and, consequently, in the metabolic byproducts. The glutamate-controlled chloride channels are outside the midgut. They have potential structural differences in the nerves and muscles, which may justify the different results found within the *Anopheles* genus. However, the authors share a strong point of view on the Fluralaner application for malaria control.

Fluralaner showed a long-lasting insecticidal effect on *An. aquasalis* mortality over 90 days. Similar long-lasting results have already been demonstrated in several studies targeting fleas and ticks [25], *Ctenocephalides felis* [50]*,* and *Demodex canis* [51]. Fluralaner fits what is considered essential for an oral insecticidal drug that can be used for malaria control [52]. Fluralaner was developed primarily for dogs against fleas and ticks [53]*,* Still, its insecticidal activity against vectors of endemic diseases has been explored, and apparently, the same efficacy as to the duration of its effect is presented in several situations.

Gomez et al*.* [38] evaluated the effect of fluralaner on *Phlebotomus papatasi* for 84 days, where the mortality remained high (60–80%) for 30 days. Bongiorno e*t al.* [26]*,* directly exposing *Phlebotomus perniciosus* to dogs medicated with fluralaner showed that the drug is 100% effective after 30 days and more than 50% effective after 84 days. Queiroga et al*.* [29]*,* evaluated fluralaner for up to 9 months and showed that in *L. longipalpis,* its effect could last for 5 months, maintaining its 100% efficacy, dropping to 72.5% in 6 months. These same authors also evaluated the impact of fluralaner in *Triatoma brasiliensis* [30], where efficacy remained at 100% over 7 months, declining to 66.4% after 8 months, 57% after 9 months, and 35% after 10 months. This study showed 100% efficacy over 90 days since *An. aquasalis* has greater sensitivity to the effect of fluralaner when compared to other species (demonstrated in LC result), it could be assumed that this efficacy would last longer. The difference in effectiveness between the different genera may be due to characteristics linked to the blood-feeding methodology used in other studies, a specific amount of meal ingestion, and the amount of blood ingested by different insects in the presence of fluralaner.

The dosage of the fluralaner is adjusted according to body weight, and the amount of the drug ingested may vary. Even though the circulating and available drug concentrations may differ in distinct animals, the drug significantly affects mortality. The durability of this effect is constant according to Queiroga et al*.* [29, 30], where several breeds of dogs of both sexes and with different weight variations were used in the experiments. It was avoided possible biases by only including dogs from the same litter, same species, and similar high body weights, bringing robustness to the analysis and the results presented here.

As previously highlighted, insecticides aim to interfere with the various developmental stages of the mosquito. The gonotrophic cycle (where the female becomes able to reproduce and lay eggs) and the sporogonic process (where the parasite completes its development inside the mosquito) are considered essential for malaria transmission [54–56]. Most of the studies cited above showed that mortality of these vectors occurred within 48 h after feeding on a fluralaner. However, this study showed that mortality occurred within 24 h. The mortality caused by fluralaner in *An. aquasalis* occurs before the female completes any of the adult developmental cycles, thus significantly decreasing the possibility of disease transmission.

Fluralaner has characteristics that allow its effectiveness and durability to be elucidated. The molecule has a high absorption capacity in several tissues, with its main concentration found in the adipose tissue, followed by the liver, kidneys, and muscles [58], as evidenced in a study published by Gomez et al*.* [38]. Kilp et al*.* [43]*,* when presenting results on the pharmacokinetics of fluralaner in dogs, make it clear that the high degree of binding of this with plasma proteins, added to liver clearance, considered low for dogs, and the high volume of distribution of liposolubility, fluralaner is highly absorbed, allowing its half-life to be prolonged. Fat is vital in how long fluralaner lasts in the animal tissue [24]. Fluralaner had a longer-lasting insecticidal effect in obese dogs compared to dogs with average weight [30]. In the trial of this study the non-drug group received blood that had its plasma extracted and reconstituted with plasma free of the drug. This group's substantial effect on *An. aquasalis* mortality may be explained by the fact that red blood cells have a lipid bilayer in their cell membrane and are most likely capable of absorbing fluralaner mainly by these fat molecules. Also, male and female dogs have different metabolisms and body fat percentages, which may explain the difference between the sexes.

Further studies, including distinct dog strains with different weights and genres, could explain the details of the actions of fluralaner. Finally, the dogs in this study always ingested fluralaner in the morning, before meals. Given that dogs got fluralaner along with their food and had better results during the length of its effects, feeding time may potentially be a factor that enhances the absorption of the drug [58].

## Conclusion

Fluralaner is a veterinary drug that has been gaining strength in studies aiming its use in the control of several endemic diseases such as malaria, where its significant effect on mortality and high half-life fit the strategies of using drugs with insecticidal action. This study shows that fluralaner is effective against the Neotropical *Anopheles aquasalis* at low concentrations. Low drug concentrations still interfere with mosquito reproduction ability when it is not lethal. It was also verified ingested fluralaner efficacy over 90 days through treated dogs. These findings incentivize the development of new research to help the several existing problems in malaria control.

### Supplementary Information

**Additional file 1: Figure S1.** Fluralaner dilution scheme for defining lethal concentrations in *Anopheles aquasalis.*

**Additional file 2: Figure S2.** Fluralaner dilution for assay on the effect on reproductive fitness of anopheles aquasalis.

**Additional file 3: **Text Information Descriptive methodology of transport and preparation of blood samples from dogs after collection.

**Additional file 4: Table S1.** The total number of deaths at different doses of Fluralaner. Dosages are expressed in µl where 2.5 µl is equivalent to 0.025 ng/ml, and 200 µl equals 2 ng/ml.

**Additional file 5: Figure S3.** Regression curve—Result of the statistical evaluation of the different mortality percentages.

**Additional file 6: Table S2.** Effects of Fluralaner on the reproductive fitness of *Anopheles aquasalis*. The table shows the mean count in absolute numbers of eggs, larvae, and pupae after drug exposure (0 = control, 1 = Fluralaner, and 9 = DMSO groups) in MFA and forced laying.

**Additional file 7: Table S3.** Survival of *Anopheles aquasalis* for 90 days fed on blood from dogs treated with Fluralaner. Values are expressed in absolute numbers as well as the percentage of mortality.

## Data Availability

This published article and its Additional files include all data generated or analyzed during this study.

## Abbreviations

ITNs: Insecticide-treated nets
IRS: Indoor residual spraying
WHO: World Health Organization
GABA: γ-Aminobutyric acid
n: Number of
BDI: Before drug ingestion
HPI: Hours post-ingestion
DPI: Days post ingestion
FMT-HVD: Fundação de Medicina Tropical Dr. Heitor Vieira Dourado
DMSO: Dimethyl sulfoxide
PBS: Phosphate-buffered saline
IMDM: Iscove culture medium
LC: Lethal concentration
MFA: Membrane feed assay
FLU: Fluralaner
CON: Control
ºC: Celsius degrees
CI: Confidence interval
IQR: Interquartile range
