# Supplementary material for: Effect of fluralaner on the biology, survival, and reproductive fitness of the neotropical malaria vector Anopheles aquasalis
Source: Malar J. 2023 Nov 7;22:337. doi: 10.1186/s12936-023-04767-0 (PMC10631211; doi:10.1186/s12936-023-04767-0)
Supplement: Supplementary file 1 — Additional file 1: Figure S1. Fluralaner dilution scheme for defining lethal concentrations in Anopheles aquasalis. [file 12936_2023_4767_MOESM1_ESM.docx]

**Additional File 1: Figure 1 - Fluralaner dilution scheme for the definition of lethal concentrations in *Anopheles aquasalis*.**

**
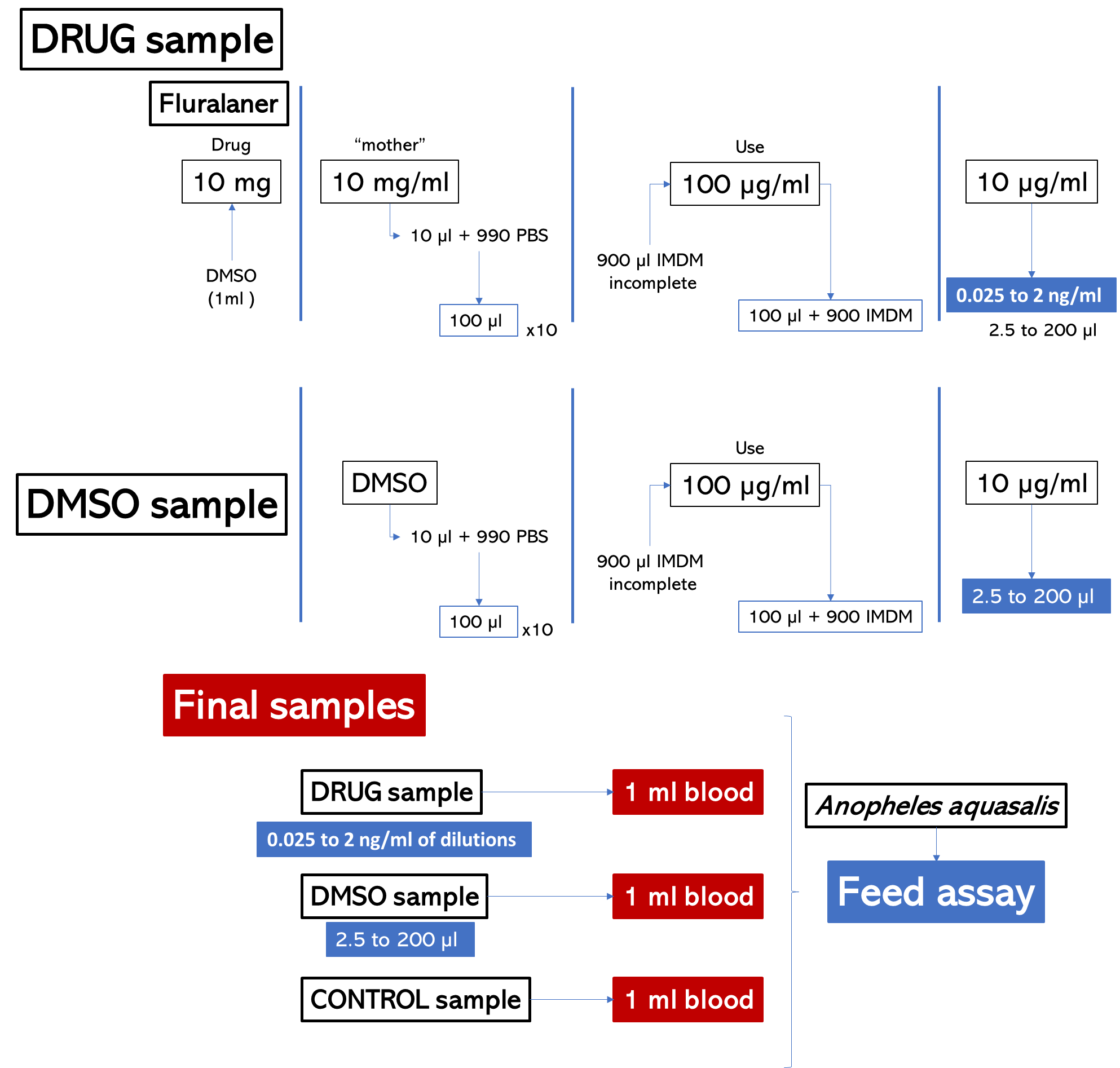
**

The squares containing the expressed values represent the aliquots
