# Supplementary material for: Effect of fluralaner on the biology, survival, and reproductive fitness of the neotropical malaria vector Anopheles aquasalis
Source: Malar J. 2023 Nov 7;22:337. doi: 10.1186/s12936-023-04767-0 (PMC10631211; doi:10.1186/s12936-023-04767-0)
Supplement: Supplementary file 2 — Additional file 2: Figure S2. Fluralaner dilution for assay on the effect on reproductive fitness of anopheles aquasalis. [file 12936_2023_4767_MOESM2_ESM.docx]

**Additional File 2: Figure 2 - Fluralaner dilutions for assay on the effect on reproductive fitness of *Anopheles aquasalis***

**
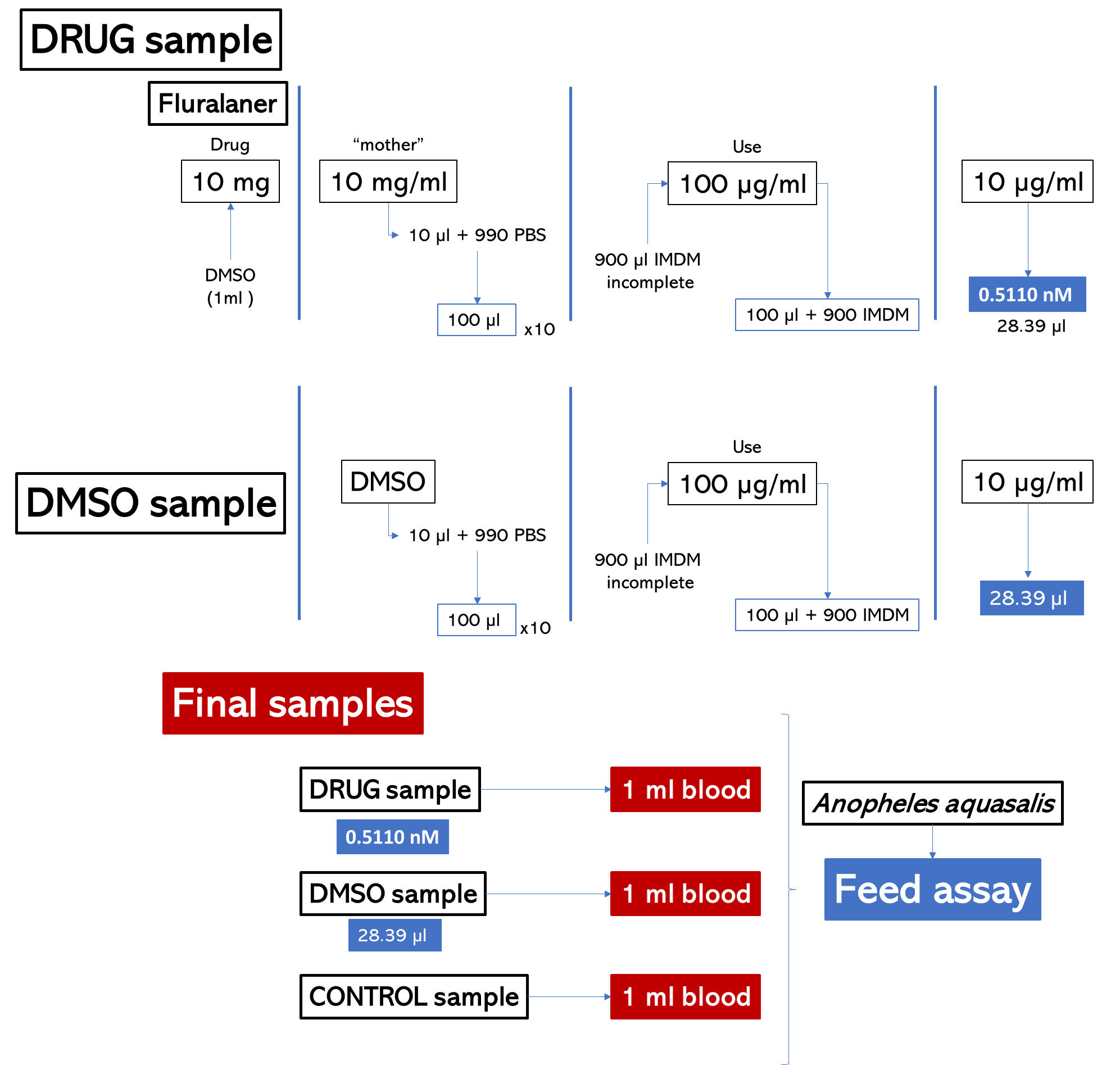
**

The squares containing the expressed values represent the aliquots
