# Supplementary material for: Effect of fluralaner on the biology, survival, and reproductive fitness of the neotropical malaria vector Anopheles aquasalis
Source: Malar J. 2023 Nov 7;22:337. doi: 10.1186/s12936-023-04767-0 (PMC10631211; doi:10.1186/s12936-023-04767-0)
Supplement: Supplementary file 3 — Additional file 3: Text Information Descriptive methodology of transport and preparation of blood samples from dogs after collection. [file 12936_2023_4767_MOESM3_ESM.docx]

**Additional File 3: Text Information - Descriptive methodology of transport and preparation of blood samples from dogs after collection**

**Collection and transport of the blood sample:** The material used for blood collection (syringe, alcohol, cotton, tourniquet) must be prepared in advance. The dog is mechanically restrained (with help of the dog owner) so that it remains immobile. The owner gets on his knees with the dog between his legs, keeping him in a quadrupedal position. With one hand, the owner hugs the dog's head and neck and, with the other, hold the chest region close to its body. The person responsible for collecting samples (veterinarian) evaluates the better possibility (jugular vein or cephalic vein). Asepsis is performed at the site before venipuncture of 9 ml of blood. If necessary, a third participant can assist in maintaining the immobilization. Specifically, in the case of collection performed in the cephalic vein, this participant is responsible for placing the tourniquet and mobilizing the foreleg. After collection, the blood is placed in a heparin tube (9ml), in a disposable glove, and in a thermos bottle previously heated internally (~37ºC) for transport to the laboratory, taking due care during the journey. Rapid locomotion is indicated for the feasibility of using the sample.

**Separation of samples for experimental infection**: Upon arrival at the laboratory, and with materials and equipment adequately prepared and sanitized, 1 ml of blood must be taken from the tube containing the blood sample and separated into a microtube (1.5 ml) being reserved for performing experimental infection (BDI Group and HPI and DPI groups. The remainder of the blood sample is reserved for centrifugation and/or reconstitution (Drug-free group).

**Centrifugation:** The remainder of the blood sample in the tube is placed in a centrifuge to separate the liquid component (plasma) from the solid component (red blood cells) at 3500 rpm, 38ºC, for 15 minutes. Using a micropipette, plasma is withdrawn and separated into microtubes containing 600 µL each. After that, an amount of RPMI relative to the total amount of blood before centrifugation is added to tube and homogenized. Again, the tube is placed in a centrifuge, repeating the specifications described above. After a second centrifugation, using a micropipette, the RPMI is removed and discarded. This process of adding the RPMI, centrifuging and discarding the RPMI is repeated two more times, leaving only the red blood cells at the bottom of the tube.

**Reconstitution:** If necessary, the plasma withdrawn and stored can be used for sample reconstitution. The microtube containing 600 µL of plasma from a previous collection and held at -80ºC must be thawed in an oven or water bath (~37ºC). With an amount of red blood cells that went through the centrifugation process described in the previous item, 400 µL of these must be removed from the tube and added to the plasma microtube. Homogenization can be carried out with the aid of a micropipette. This reconstituted blood sample can be used for experimental infection (Drug-free group)
