# Supplementary material for: Effect of fluralaner on the biology, survival, and reproductive fitness of the neotropical malaria vector Anopheles aquasalis
Source: Malar J. 2023 Nov 7;22:337. doi: 10.1186/s12936-023-04767-0 (PMC10631211; doi:10.1186/s12936-023-04767-0)
Supplement: Supplementary file 4 — Additional file 4: Table S1. The total number of deaths at different doses of Fluralaner. Dosages are expressed in µl where 2.5 µl is equivalent to 0.025 ng/ml, and 200 µl equals 2 ng/ml. [file 12936_2023_4767_MOESM4_ESM.docx]

**Additional File 4: Table 1 - Total number of deaths at different doses of fluralaner.** Dosages are expressed in µl where 2.5 µl is equivalent to 0.025 ng/ml and 200 µl is equivalent to 2 ng/ml.

| **dose** | **dead** | **total** |
| --- | --- | --- |
| 0.0 | 34 | 662 |
| 2.5 | 0 | 32 |
| 5.0 | 2 | 143 |
| 10.0 | 2 | 93 |
| 15.0 | 3 | 131 |
| 20.0 | 9 | 188 |
| 25.0 | 29 | 364 |
| 30.0 | 2 | 108 |
| 35.0 | 11 | 100 |
| 40.0 | 10 | 242 |
| 50.0 | 43 | 328 |
| 60.0 | 12 | 164 |
| 70.0 | 9 | 101 |
| 80.0 | 10 | 80 |
| 90.0 | 8 | 89 |
| 100.0 | 71 | 236 |
| 120.0 | 7 | 68 |
| 150.0 | 191 | 345 |
| 180.0 | 7 | 71 |
| 200.0 | 153 | 238 |

Dosages are expressed in µl where 2.5 µl is equivalent to 0.025 ng/ml and 200 µl is equivalent to 2 ng/ml.
