# Supplementary material for: Effect of fluralaner on the biology, survival, and reproductive fitness of the neotropical malaria vector Anopheles aquasalis
Source: Malar J. 2023 Nov 7;22:337. doi: 10.1186/s12936-023-04767-0 (PMC10631211; doi:10.1186/s12936-023-04767-0)
Supplement: Supplementary file 5 — Additional file 5: Figure S3. Regression curve—Result of the statistical evaluation of the different mortality percentages. [file 12936_2023_4767_MOESM5_ESM.docx]

**Additional File 5: Figure 3 - Regression curve -** Result of the statistical evaluation on the different mortality percentages


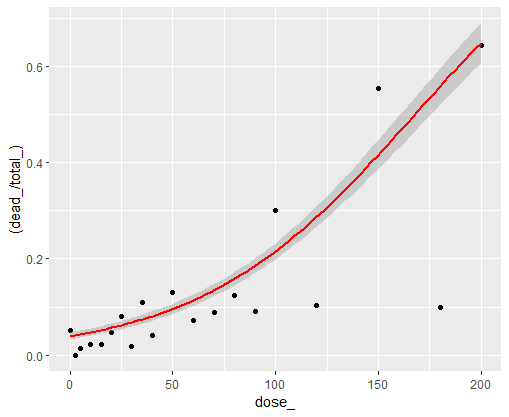


The percentage of deaths is expressed on the Y axis and the dosage on the X axis, where 200 µl is equivalent to 2 ng/ml and 0 is the control group.
