# Supplementary material for: Effect of fluralaner on the biology, survival, and reproductive fitness of the neotropical malaria vector Anopheles aquasalis
Source: Malar J. 2023 Nov 7;22:337. doi: 10.1186/s12936-023-04767-0 (PMC10631211; doi:10.1186/s12936-023-04767-0)
Supplement: Supplementary file 6 — Additional file 6: Table S2. Effects of Fluralaner on the reproductive fitness of Anopheles aquasalis. The table shows the mean count in absolute numbers of eggs, larvae, and pupae after drug exposure (0 = control, 1 = Fluralaner, and 9 = DMSO groups) in MFA and forced laying. [file 12936_2023_4767_MOESM6_ESM.docx]

**Additional File 6: Table 2 - Effects of fluralaner on the reproductive fitness of *Anopheles aquasalis***. The table shows the mean count in absolute numbers of eggs, larvae, and pupae after drug exposure (0=control, 1=Fluralaner, and 9= DMSO groups) in MFA and forced laying

| **Assay** | **group** | **id** | **eggs** | **larvae** | **pulpae** |
| --- | --- | --- | --- | --- | --- |
| 1 | 0 | 1 | 232 | 132 | 124 |
| 1 | 0 | 2 | 146 | 3 | 2 |
| 1 | 0 | 3 | 278 | 153 | 152 |
| 1 | 0 | 4 | 245 | 128 | 102 |
| 1 | 0 | 5 | 277 | 155 | 154 |
| 1 | 0 | 6 | 153 | 81 | 81 |
| 1 | 0 | 7 | 206 | 101 | 93 |
| 1 | 0 | 8 | 264 | 138 | 134 |
| 1 | 0 | 9 | 233 | 117 | 100 |
| 1 | 0 | 10 | 245 | 127 | 124 |
| 1 | 0 | 11 | 245 | 128 | 124 |
| 1 | 0 | 12 | 220 | 112 | 106 |
| 1 | 0 | 13 | 202 | 110 | 80 |
| 1 | 0 | 14 | 217 | 91 | 82 |
| 1 | 0 | 15 | 276 | 148 | 146 |
| 1 | 0 | 16 | 240 | 133 | 117 |
| 1 | 0 | 17 | 234 | 116 | 103 |
| 1 | 0 | 18 | 0 | 0 | 0 |
| 1 | 0 | 19 | 0 | 0 | 0 |
| 1 | 0 | 20 | 0 | 0 | 0 |
| 1 | 1 | 1 | 115 | 65 | 64 |
| 1 | 1 | 2 | 166 | 86 | 84 |
| 1 | 1 | 3 | 8 | 5 | 5 |
| 1 | 1 | 4 | 116 | 58 | 56 |
| 1 | 1 | 5 | 62 | 56 | 34 |
| 1 | 1 | 6 | 132 | 35 | 59 |
| 1 | 1 | 7 | 95 | 62 | 56 |
| 1 | 1 | 8 | 0 | 59 | 0 |
| 1 | 1 | 9 | 0 | 0 | 0 |
| 1 | 1 | 10 | 0 | 0 | 0 |
| 1 | 1 | 11 | 0 | 0 | 0 |
| 1 | 1 | 12 | 0 | 0 | 0 |
| 1 | 1 | 13 | 0 | 0 | 0 |
| 1 | 1 | 14 | 0 | 0 | 0 |
| 1 | 1 | 15 | 0 | 0 | 0 |
| 1 | 1 | 16 | 0 | 0 | 0 |
| 1 | 1 | 17 | 0 | 0 | 0 |
| 1 | 1 | 18 | 0 | 0 | 0 |
| 1 | 1 | 19 | 0 | 0 | 0 |
| 1 | 1 | 20 | 0 | 0 | 0 |
| 1 | 9 | 1 | 249 | 124 | 116 |
| 1 | 9 | 2 | 219 | 119 | 108 |
| 1 | 9 | 3 | 278 | 133 | 122 |
| 1 | 9 | 4 | 175 | 93 | 87 |
| 1 | 9 | 5 | 226 | 115 | 14 |
| 1 | 9 | 6 | 155 | 3 | 3 |
| 1 | 9 | 7 | 243 | 113 | 106 |
| 1 | 9 | 8 | 285 | 95 | 91 |
| 1 | 9 | 9 | 264 | 145 | 141 |
| 1 | 9 | 10 | 247 | 127 | 117 |
| 1 | 9 | 11 | 148 | 72 | 65 |
| 1 | 9 | 12 | 194 | 97 | 93 |
| 1 | 9 | 13 | 230 | 119 | 115 |
| 1 | 9 | 14 | 237 | 125 | 124 |
| 1 | 9 | 15 | 284 | 146 | 145 |
| 1 | 9 | 16 | 218 | 139 | 134 |
| 1 | 9 | 17 | 0 | 0 | 0 |
| 1 | 9 | 18 | 0 | 0 | 0 |
| 1 | 9 | 19 | 0 | 0 | 0 |
| 1 | 9 | 20 | 0 | 0 | 0 |
| 2 | 0 | 1 | 123 | 70 | 66 |
| 2 | 0 | 2 | 130 | 83 | 69 |
| 2 | 0 | 3 | 97 | 61 | 61 |
| 2 | 0 | 4 | 106 | 70 | 68 |
| 2 | 0 | 5 | 173 | 93 | 82 |
| 2 | 0 | 6 | 85 | 53 | 50 |
| 2 | 0 | 7 | 132 | 98 | 98 |
| 2 | 0 | 8 | 211 | 126 | 114 |
| 2 | 0 | 9 | 95 | 56 | 52 |
| 2 | 0 | 10 | 110 | 52 | 43 |
| 2 | 0 | 11 | 103 | 70 | 65 |
| 2 | 0 | 12 | 116 | 88 | 85 |
| 2 | 0 | 13 | 12 | 0 | 0 |
| 2 | 0 | 14 | 96 | 43 | 28 |
| 2 | 0 | 15 | 0 | 0 | 0 |
| 2 | 0 | 16 | 0 | 0 | 0 |
| 2 | 0 | 17 | 0 | 0 | 0 |
| 2 | 0 | 18 | 0 | 0 | 0 |
| 2 | 0 | 19 | 0 | 0 | 0 |
| 2 | 0 | 20 | 0 | 0 | 0 |
| 2 | 1 | 1 | 136 | 39 | 30 |
| 2 | 1 | 2 | 100 | 38 | 36 |
| 2 | 1 | 3 | 89 | 41 | 40 |
| 2 | 1 | 4 | 81 | 11 | 10 |
| 2 | 1 | 5 | 28 | 2 | 1 |
| 2 | 1 | 6 | 0 | 0 | 0 |
| 2 | 1 | 7 | 0 | 0 | 0 |
| 2 | 1 | 8 | 0 | 0 | 0 |
| 2 | 1 | 9 | 0 | 0 | 0 |
| 2 | 1 | 10 | 0 | 0 | 0 |
| 2 | 1 | 11 | 0 | 0 | 0 |
| 2 | 1 | 12 | 0 | 0 | 0 |
| 2 | 1 | 13 | 0 | 0 | 0 |
| 2 | 1 | 14 | 0 | 0 | 0 |
| 2 | 1 | 15 | 0 | 0 | 0 |
| 2 | 1 | 16 | 0 | 0 | 0 |
| 2 | 1 | 17 | 0 | 0 | 0 |
| 2 | 1 | 18 | 0 | 0 | 0 |
| 2 | 1 | 19 | 0 | 0 | 0 |
| 2 | 1 | 20 | 0 | 0 | 0 |
| 2 | 9 | 1 | 79 | 73 | 62 |
| 2 | 9 | 2 | 116 | 82 | 75 |
| 2 | 9 | 3 | 59 | 38 | 38 |
| 2 | 9 | 4 | 111 | 61 | 60 |
| 2 | 9 | 5 | 89 | 77 | 74 |
| 2 | 9 | 6 | 107 | 90 | 89 |
| 2 | 9 | 7 | 121 | 83 | 77 |
| 2 | 9 | 8 | 95 | 62 | 62 |
| 2 | 9 | 9 | 67 | 36 | 34 |
| 2 | 9 | 10 | 190 | 120 | 116 |
| 2 | 9 | 11 | 163 | 70 | 65 |
| 2 | 9 | 12 | 124 | 61 | 52 |
| 2 | 9 | 13 | 83 | 50 | 46 |
| 2 | 9 | 14 | 0 | 0 | 0 |
| 2 | 9 | 15 | 0 | 0 | 0 |
| 2 | 9 | 16 | 0 | 0 | 0 |
| 2 | 9 | 17 | 0 | 0 | 0 |
| 2 | 9 | 18 | 0 | 0 | 0 |
| 2 | 9 | 19 | 0 | 0 | 0 |
| 2 | 9 | 20 | 0 | 0 | 0 |
| 3 | 0 | 1 | 226 | 131 | 109 |
| 3 | 0 | 2 | 246 | 138 | 129 |
| 3 | 0 | 3 | 182 | 120 | 17 |
| 3 | 0 | 4 | 238 | 128 | 114 |
| 3 | 0 | 5 | 169 | 107 | 96 |
| 3 | 0 | 6 | 187 | 112 | 89 |
| 3 | 0 | 7 | 143 | 72 | 68 |
| 3 | 0 | 8 | 174 | 78 | 65 |
| 3 | 0 | 9 | 172 | 106 | 100 |
| 3 | 0 | 10 | 148 | 83 | 79 |
| 3 | 0 | 11 | 176 | 92 | 78 |
| 3 | 0 | 12 | 229 | 113 | 91 |
| 3 | 0 | 13 | 151 | 95 | 88 |
| 3 | 0 | 14 | 182 | 99 | 92 |
| 3 | 0 | 15 | 243 | 123 | 96 |
| 3 | 0 | 16 | 221 | 116 | 108 |
| 3 | 0 | 17 | 0 | 0 | 0 |
| 3 | 0 | 18 | 0 | 0 | 0 |
| 3 | 0 | 19 | 0 | 0 | 0 |
| 3 | 0 | 20 | 0 | 0 | 0 |
| 3 | 1 | 1 | 103 | 68 | 64 |
| 3 | 1 | 2 | 65 | 37 | 33 |
| 3 | 1 | 3 | 164 | 77 | 48 |
| 3 | 1 | 4 | 114 | 80 | 79 |
| 3 | 1 | 5 | 122 | 2 | 2 |
| 3 | 1 | 6 | 135 | 60 | 57 |
| 3 | 1 | 7 | 0 | 0 | 0 |
| 3 | 1 | 8 | 0 | 0 | 0 |
| 3 | 1 | 9 | 0 | 0 | 0 |
| 3 | 1 | 10 | 0 | 0 | 0 |
| 3 | 1 | 11 | 0 | 0 | 0 |
| 3 | 1 | 12 | 0 | 0 | 0 |
| 3 | 1 | 13 | 0 | 0 | 0 |
| 3 | 1 | 14 | 0 | 0 | 0 |
| 3 | 1 | 15 | 0 | 0 | 0 |
| 3 | 1 | 16 | 0 | 0 | 0 |
| 3 | 1 | 17 | 0 | 0 | 0 |
| 3 | 1 | 18 | 0 | 0 | 0 |
| 3 | 1 | 19 | 0 | 0 | 0 |
| 3 | 1 | 20 | 0 | 0 | 0 |
| 3 | 9 | 1 | 273 | 157 | 150 |
| 3 | 9 | 2 | 283 | 128 | 129 |
| 3 | 9 | 3 | 280 | 164 | 119 |
| 3 | 9 | 4 | 251 | 106 | 113 |
| 3 | 9 | 5 | 159 | 104 | 98 |
| 3 | 9 | 6 | 202 | 113 | 99 |
| 3 | 9 | 7 | 142 | 70 | 68 |
| 3 | 9 | 8 | 112 | 69 | 64 |
| 3 | 9 | 9 | 235 | 115 | 100 |
| 3 | 9 | 10 | 187 | 102 | 78 |
| 3 | 9 | 11 | 196 | 96 | 79 |
| 3 | 9 | 12 | 202 | 105 | 99 |
| 3 | 9 | 13 | 213 | 112 | 89 |
| 3 | 9 | 14 | 171 | 103 | 90 |
| 3 | 9 | 15 | 189 | 124 | 101 |
| 3 | 9 | 16 | 176 | 114 | 111 |
| 3 | 9 | 17 | 0 | 0 | 0 |
| 3 | 9 | 18 | 0 | 0 | 0 |
| 3 | 9 | 19 | 0 | 0 | 0 |
| 3 | 9 | 20 | 0 | 0 | 0 |

The table shows the mean count in absolute numbers of eggs, larvae and pupae after drug exposure (0=control, 1=Fluralaner and 9= DMSO groups) in MFA and forced laying.
