# Supplementary material for: Effect of fluralaner on the biology, survival, and reproductive fitness of the neotropical malaria vector Anopheles aquasalis
Source: Malar J. 2023 Nov 7;22:337. doi: 10.1186/s12936-023-04767-0 (PMC10631211; doi:10.1186/s12936-023-04767-0)
Supplement: Supplementary file 7 — Additional file 7: Table S3. Survival of Anopheles aquasalis for 90 days fed on blood from dogs treated with Fluralaner. Values are expressed in absolute numbers as well as the percentage of mortality. [file 12936_2023_4767_MOESM7_ESM.docx]

**Additional File 7: Table 3 - Survival of *Anopheles aquasalis* for 90 days fed on blood from dogs treated with fluralaner**. Values are expressed in absolute numbers as well as the percentage of mortality

| **Day** | **Dead** | **Alive** | **Total** | **Percentage** |
| --- | --- | --- | --- | --- |
| 0 | 20 | 590 | 610 | 3.28 |
| 24 | 485 | 0 | 485 | 100.00 |
| 7 | 465 | 0 | 465 | 100.00 |
| 14 | 603 | 0 | 603 | 100.00 |
| 21 | 569 | 0 | 569 | 100.00 |
| 28 | 578 | 0 | 578 | 100.00 |
| 60 | 525 | 0 | 525 | 100.00 |
| 90 | 492 | 0 | 492 | 100.00 |
|  |  |  |  |  |
| Drug Free |  |  |  |  |
| 24 | 250 | 0 | 250 | 100.00 |
| 7 | 221 | 0 | 221 | 100.00 |
| 14 | 278 | 0 | 278 | 100.00 |
| 21 | 217 | 0 | 217 | 100.00 |
| 28 | 352 | 0 | 352 | 100.00 |
| 60 | 56 | 187 | 243 | 23.05 |
| 90 | 39 | 233 | 272 | 14.34 |

Drug-free group by sex

| DF Female |  |  |  |  |
| --- | --- | --- | --- | --- |
| 24 | 113 | 0 | 113 | 100.00 |
| 7 | 101 | 0 | 101 | 100.00 |
| 14 | 137 | 0 | 137 | 100.00 |
| 21 | 132 | 0 | 132 | 100.00 |
| 28 | 200 | 0 | 200 | 100.00 |
| 60 | 0 | 107 | 107 | 0.00 |
| 90 | 10 | 141 | 151 | 6.62 |
|  |  |  |  |  |
| DF Male |  |  |  |  |
| 24 | 137 | 0 | 137 | 100.00 |
| 7 | 120 | 0 | 120 | 100.00 |
| 14 | 141 | 0 | 141 | 100.00 |
| 21 | 85 | 0 | 85 | 100.00 |
| 28 | 152 | 0 | 152 | 100.00 |
| 60 | 56 | 80 | 136 | 41.18 |
| 90 | 29 | 92 | 121 | 23.97 |

Values are expressed in absolute numbers as well as the percentage of mortality.

DF – Drug-free groups
